# Challenge of Retinoblastoma in Mexico in 2020: Perspectives and Solutions

**DOI:** 10.1155/2020/1953602

**Published:** 2020-08-10

**Authors:** Lucas A. Garza-Garza, Raúl E. Ruiz-Lozano, Genaro Rebolledo-Méndez, Ismael Ibarra-Nava, Héctor J. Morales-Garza, David Ancona-Lezama

**Affiliations:** ^1^Tecnologico de Monterrey, School of Medicine and Health Sciences, Ocular Oncology Service at Institute of Ophthalmology and Visual Sciences, Hospital Zambrano-Hellion, San Pedro Garza Garcia, Nuevo León, Mexico; ^2^Writing Lab, TecLabs, Vicerrectoria de Investigación y Transferencia de Tecnología, Tecnologico de Monterrey, Monterrey, Mexico; ^3^Department of Preventive Medicine and Public Health, Faculty of Medicine, Universidad Autónoma de Nuevo León, Monterrey, Mexico

## Abstract

Early diagnosis and positive outcomes of retinoblastoma in childhood have been positively correlated with the economic wealth of high-income countries (HICs) worldwide. Adequate curability and survival rates, adherence to treatment, presence of poor prognostic initial clinical signs, and metastatic disease at diagnosis appear to have a less favorable picture in low-income countries (LICs). However, this is not always the case. An example is Argentina, where disease-free survival rates of retinoblastoma are notably higher than expected when taking into consideration its economic situation. Unfortunately, as in other Latin American LICs, retinoblastoma outcomes in Mexico are worrisome. Interestingly, the Human Development Index (HDI) in Mexico varies widely between its different geographical regions. While in some states, the HDI resembles those of high-income countries, and in others, the opposite is observed. A unifying picture of Mexico's developmental status, health resources, indicators, and other factors possibly influencing outcomes in retinoblastoma is currently unavailable. The present review explores the previously mentioned factors in Mexico and compares them to other countries. Additionally, it recommends solutions or enhancements where possible.

## 1. Introduction

Retinoblastoma (Rb) is the most frequent intraocular malignancy of childhood [1]. Global incidence rates remain constant, with approximately 9,000 new cases every year [2]. However, a higher mortality rate is registered in Asia and Africa, where between 40 and 70% of children with Rb die, compared with only 3–5% in some high-income countries (HICs) [2]. Still, selected countries have recently reported better outcomes in Rb, including India, with a survival rate of 75.2–92% and Taiwan with 80.9% [3, 4]. In Mexico, mortality rates range from 9.1–16%, which resemble those observed in most Latin American countries [5]. Interestingly, despite being a middle-income country (MIC), Argentina has a Rb mortality rate of 7% [6]. Survival rates and preservation of vision are highly dependent on early diagnosis and treatment. Unfortunately, health system constraints, poor education, and unequal access to care in low- and middle-income countries (LMICs) lead to delays in the diagnosis and treatment of Rb. As in maternal survival rates, survival in Rb represents a growing marker of a nation's development [1]. Previous Mexican studies report an estimated incidence of 5.6 cases Rb cases per million children, which slightly surpasses the incidence reported in the United States (US; 3.9 cases per million) [7].

Another indicator of adequate Rb management is the presenting signs and symptoms of the disease. Initial clinical Rb findings include leukocoria, strabismus, decreased visual acuity, ocular inflammation, glaucoma, hyphema, buphthalmos, proptosis, and massive extraocular extension [8, 9]. While leukocoria, strabismus, and decreased visual acuity represent early-stage disease manifestations, proptosis, glaucoma, and congestive intraocular signs are prevalently seen in advanced stages of Rb [5]. Previously reported presenting signs and symptoms from Rb in Mexico suggest a better scenario than those reported in other LMICs. In Ethiopia, proptosis (53.7%) was the most frequent presenting sign of Rb [8]. A broad systematic review of Rb patients in Mexico reported leukocoria (79.3%) and strabismus (50%) as the most common presenting findings [5]. Signs of advanced-stage disease such as glaucoma (0.7%) or proptosis (3.3%) were uncommon [5]. The latter might imply that the reduced survival rates of Rb in Mexico, compared with those in HICs, are explained by poor access to therapy and adequate follow-up, rather than a decreased success in early diagnosis.

Metastatic disease at diagnosis, treatment refusal, and therapy abandonment rates are high among LICs [6]. In Mexico, 7% of Rb cases present with metastatic disease at diagnosis, while a statistic for treatment refusal is not available [6]. In Latin America, up to 26% of cancer patients abandon their therapy [5]. On the other hand, overall treatment abandonment in Mexican cancer patients has been reported as significantly lower, ranging from 4.5 to 18% [10–12]. Moreover, delay in diagnosis, which limits treatment alternatives and therefore, curability, has been reported to be about 6–7 months in Mexico [5]. In contrary to other Latin American countries, Argentina has a low incidence of metastatic disease at diagnosis and treatment refusal, with rates of 3% and 2%, respectively [6].

In Mexico, where cancer is the leading cause of death in children age 5–9 years and the fourth in those younger than 20 years, a closer inspection of the current situation is fundamental [5]. Moreover, a closer assessment of available data reveals a potentially overlooked geographic difference between Mexican federal states. Such is the case of the southern state of Chiapas, which reports a higher incidence rate of Rb (21.4 cases per million children every year) compared with the national average [7]. Similarly, African countries present rates as high as 21 cases per million children [1]. Geographical differences in mortality, presenting symptoms, metastatic disease at diagnosis, lag-time for diagnosis, and treatment refusal might exist in Mexico, but stratified data by state are currently not available.

Previous studies have explored the relationship between the developmental status of a nation and the survival rate and other outcomes (lag-time to diagnosis and treatment compliance) of Rb [13]. The status, survival rates, and disease outcomes in Mexico have been described and compared with those of other countries [6, 13]. Nevertheless, a detailed picture of the country's developmental status and its indicators, health programs, availability of health specialists and centers, and other factors influencing Rb and outcomes is still unavailable. This detailed overview explores the HDI situation in Mexico to throw light on Rb indicators and recommends solutions (Table 1) or enhancements where possible.

## 2. Insufficient Data of Retinoblastoma in Mexico

As previously stated, Mexico has polarizing differences between its geographical regions. The abovementioned economic, social, and health disparity that Mexico is facing renders different panoramas when managing Rb. Unfortunately, previous Rb reports in Mexico fail to provide complete demographic data concerning the patients' origins and socioeconomic status, making subgroup analysis unreliable [5, 14, 15]. As previous authors have suggested, creating a national registry to document, follow, and analyze Rb cases is of utmost importance [5, 14]. Previously, a multicenter report described the characteristics, outcomes, and treatment of Rb in Mexico, comprising 16 different institutions [14]. In addition, a multicenter national registry was created with the support of 27 institutions but has not yet encompassed all Mexican centers that currently treat Rb [16]. Continuing efforts in this direction are mandatory, so one day, a full Nation-wide registry can be created. The latter will allow us to discern which regions and which strategies should be prioritized in order to improve the outcomes in Rb.

## 3. Human Development Index (HDI) as an Indicator for Retinoblastoma

HDI arose from a United Nations' collaborative effort as a tool for measuring human development in every country in the world [17]. This index integrates three essential aspects of a population: those related to a long and healthy life (health), acquired knowledge (education), and decent standard of living (economics) [17]. This index has been previously linked to outcomes in retinoblastoma, such as disease-free survival, early detection, and globe salvage [1]. However, considering a country might undermine the differences within its regions, especially those poorly represented by the mean National HDI. The most recent report measured Mexico's HDI from 2,456 municipalities and reported a 2.5 times difference between the highest and lowest ranks. Additionally, the most polarized HDI component among municipalities is education (with a difference superior to 70%), followed by health (difference of 65%). The reported difference in HDI between the highest and lowest Mexican municipalities is analogous to that between the Netherlands and Ghinea-Bissau [18].

## 4. Specialist Availability and Third-Level Centers in Mexico

A distribution disparity exists between the number of ophthalmologists per federal state in Mexico, with the lowest state having only 15 and the highest 894 registered ophthalmologists (unpublished data) [19]. In addition, the number of hospitals also differ importantly per federal state [20]. In 2014, the states with the most hospitals had 7 per 100,000 people, and those with the least had 1 per 100,000 people [20]. Besides, the number of surgical centers per federal state ranges from 14 per 100,000 people (Mexico City) to 4 per 100,000 people (Chiapas) [20]. Hospitals and surgical centers have increased in number in many federal states in Mexico in the last decade; however, most of this development has been noted in states that previously had the most hospitals and surgical centers [20]. Furthermore, most of the new hospitals and surgical centers created belong to the private sector, making it less approachable to the most vulnerable population [14]. While a formal statistic on the number of institutions that treat patients with Rb or other pediatric cancers is unavailable, previously published data suggest that the number of specialists is low compared with other countries [11]. For example, only 183 board-certified pediatric oncologists are available in Mexico as a whole, while 2079 exist in the United States [11]. Additionally, only six ophthalmologists with formal ocular oncology training are available in the country, and not all of them currently treat Rb (Ancona-Lezama, personal communication). Registered nurses, another essential element in the care of pediatric cancer patients, are also less available than in other countries (Mexico, 1.57 nurses/1,000 individuals vs. Canada, and 6.69 nurses/1,000 individuals) [11]. Finally, specific therapies used in advanced stages of Rb, like intraarterial chemotherapy, are currently available only in a few certified centers in the country (Ancona-Lezama, personal communication).

## 5. Other Factors Influencing Retinoblastoma Outcomes in Mexico

Previous authors have hypothesized that other factors, apart from mean national HDI, could affect RB care outcomes, including awareness of the medical population and awareness campaigns, maternal-child health programs, and universal health care (or at least free childhood care) [13]. While some awareness campaigns in Mexico targeting Rb have been undertaken, other studies report that the general medical population performs poorly when tested on the topic [13, 21]. Previous studies report that medical graduates have a strong tendency to fail in both recognizing leukocoria as an early sign of Rb and acknowledging this disease as a life-threatening cancer [22]. Education concerning Rb awareness should be implemented during medical formation and reinforced amongst physicians, especially pediatricians and ophthalmologists.

On the other hand, maternal-child health programs have been strongly supported in Mexico. For example, the federal program “Control del Niño Sano” is a free-of-charge health program where every child younger than five years has access to periodic consults where nutrition, neurological development, accident prevention, and detection of congenital and infectious diseases are monitored and reinforced [23]. Mexico achieved universal health care after the introduction of “Seguro Popular” in 2003, which aimed to cover people without social security or private insurance at the primary, secondary, and tertiary levels [24]. Seguro Popular financing included a combination of federal and state funds coming from general taxes and, in some cases, personal or family premium payments. Furthermore, care provision and resource administration were the responsibility of each state. This program enlisted the most critical diseases from which the general population would be universally covered, including Rb [25]. In 2019, this public scheme was replaced by the Instituto de Salud para el Bienestar (INSABI), which aims to provide health care to the same target population but free of charge [26]. Previous statements from the Mexican government claim that diseases previously covered by “Seguro Popular,” such as Rb, will still be covered by the new system “INSABI” [27]. Challenges remain as the health system transitions to this new scheme. One of the most important challenges being the centralization of the administration and coordination of resources by the federal government, which some states have refused. In fact, 23 out of 32 (72%) states signed the agreement to release the responsibility [28]. The nine remaining states will have to independently ensure that the uninsured population receives the care they need, free of charge.

## 6. Possible Solutions for Improving Retinoblastoma Outcomes in Mexico

### 6.1. Overcoming Geographical Disparities

As previously stated, HDIs, human and technological resources in healthcare, and access and availability of effective treatments vary significantly across regions in Mexico. While an increase in the HDI homogeneity of the country's different geographical regions may be decade consuming, it is a task that must be reinforced. In addition, a redirection and concentration of hospital and surgical centers' building resources to the states with the lowest number should be undertaken. The current political administration in Mexico has announced its intentions to provide health infrastructure and resources for the poorest population sectors [29]. On the other hand, a redistribution of the available specialists to provide an even number per federal state seems unlikely. A more plausible alternative could be to provide access to telemedicine to those states with insufficient specialists or those who need subspecialists (ocular oncologists). Telemedicine has been previously implemented in Rb care in LICs with successful results [30, 31]. Mexico's disparity in the level of care could be used to its advantage by encouraging high-income federal states to provide advice and diagnosis using telemedicine applications in those with low-income. While programs for telemedicine in ophthalmology and other areas exist in Mexico, no such program has been implemented with Rb as a target [32]. We suggest such a program could represent a low-cost solution to bridge the previously discussed gap of ophthalmologists between states.

Promoting universal eye health coverage could reduce health disparities across states and regions [33]. To achieve this, investment in primary health care (PHC) is essential, especially where resources are scarce and where there is a lack of a more specialized health workforce. PHC is a highly effective and efficient way to address health challenges and people's health needs [34]. VISION 2020 is a global initiative led by the World Health Organization (WHO), which recommends as a key strategy the “promotion of eye health and the provision of basic preventive and curative treatment for common eye disorders” as part of PHC [35]. This initiative would also require strengthening referral systems to secondary and tertiary levels that can provide effective treatments if needed.

### 6.2. Increasing Awareness and Medical Education

Awareness and health education campaigns for Rb and other pediatric cancers have been previously undertaken in Mexico [13, 36]. In 2003, the Grupo Mexicano de Retinoblastoma (RtbMex) program was created to coordinate efforts among groups aiming to improve care for patients with Rb. Part of the strategy implemented included a massive public education campaign in public places, schools, and health care centers. Furthermore, health care professionals were also educated with seminars and printed materials [16]. Despite the efforts of RtbMex, knowledge among healthcare professionals remains deficient, especially in southern Mexico [21]. The maintenance and stimulation of these campaigns should be encouraged, for some evidence exists that they could reduce the number of advanced cases [37]. Furthermore, more effective awareness campaigns and capacity-building programs targeting health professionals should be implemented, especially in poorer states that lack high-quality medical education. The latter results in insufficient knowledge of Rb among general physicians [22]. These programs should be integrated during both the physicians' formation and as a form of continuous medical education. All Mexican medical students undergo a mandatory year of social service following the termination of their internship. Most social service graduates are assigned to the most marginalized municipalities where physician availability is scarce or nonexistent. A significant percentage use that time as preparation for the residency matching exam called “Examen Nacional de Aspirantes a Residencias Médicas” (ENARM). An optional online course or certification could be widely distributed among them with curricular incentives provided by Mexico's top universities.

### 6.3. Twinning

Twinning is an innovative approach between health institutions used to improve health service delivery [38]. Partnerships between health institutions using this approach usually occur between those in HICs and those in LMICs. The main goals are to develop pediatric cancer units, foster alliances among different stakeholders, improve medical practices, and increase participation in collaborative research projects. Twinning projects in Central and South America and the Middle East have resulted in improved outcomes for children with cancer [16]. In Mexico, this approach could be implemented within the country by creating partnerships between institutions that are more specialized in cancer care and institutions that do not have enough resources to address the healthcare needs of patients with Rb.

## 7. Conclusions

Retinoblastoma continues to be a big challenge in Mexico. Moreover, it may pose an even greater challenge in specific regions of the country. Mortality, survival rate, presenting symptoms, delay to diagnosis, metastatic disease at diagnosis, and therapy abandonment are clinical indicators that should be monitored at the national and subnational levels to assess for Rb management advancements. Additionally, health inequities and disparities, shortage of research and data, insufficient and unequal specialists' distribution, and suboptimal medical education of Rb are pressing main challenges that need to be readily addressed to improve patient outcomes and enhance the quality of life. To accomplish this, significant investments in research, education, and human and technological resources will need to be implemented in regions with higher needs to bridge the current health gaps in Rb.

## Figures and Tables

**Table 1:** Challenges and possible solutions for improving retinoblastoma outcomes in Mexico.

Challenge	Possible solutions
Differences in retinoblastoma (Rb) incidence, HDI, health resources, and professional availability among federal states	1. Promoting universal eye health coverage 2. Specific direction of health resources to the most vulnerable states 3. Twinning and telemedicine

Insufficient data and lack of a subgroup analysis of Rb outcomes per federal state	1. Encompassing all public and private institutions from different federal states in existing or new national registries 2. Modification of registries to allow for complete demographic data and subsequent subgroup analysis

Insufficient medical knowledge, delayed Rb diagnosis, metastatic disease at diagnosis, and cancer therapy abandonment	1. Strengthening Rb programs in general medicine academic formation 2. Maintenance and stimulation of Rb awareness campaigns for the medical and general population 3. Promoting universal eye health coverage 4. Vigilance and timewise intervention in cases with risk of therapy abandonment

Maternal-fetal programs and new universal health coverage	1. An excellent program (“control del niño sano”) already exists to cover child health from birth to 5 years old; it should be maintained and strengthened 2. Previous universal health programs have successfully covered Rb; this coverage should be maintained and strengthened
